# Hypervitaminosis D Secondary to a *CYP24A1* Loss‐of‐Function Mutation: An Unusual Cause of Hypercalcemia in Two Siblings

**DOI:** 10.1002/jbm4.10788

**Published:** 2023-08-08

**Authors:** Lucy Collins, Emma Boehm, Catherine Luxford, Roderick Clifton‐Bligh, Vivian Grill

**Affiliations:** ^1^ Department of Endocrinology and Diabetes Western Health Melbourne Victoria Australia; ^2^ University of Melbourne Melbourne Victoria Australia; ^3^ Royal North Shore Hospital Kolling Institute St Leonards New South Wales Australia; ^4^ Department of Endocrinology Royal North Shore Hospital St Leonards New South Wales Australia

**Keywords:** CELL/TISSUE SIGNALING – ENDOCRINE PATHWAYS, DISORDERS OF CALCIUM/PHOSPHATE METABOLISM, OTHER, PTH/VIT D/FGF23, THERAPEUTICS

## Abstract

Hypervitaminosis D as a cause of hypercalcemia may be due to vitamin D intoxication, granulomatous diseases, or abnormalities of vitamin D metabolism. The *CYP24A1* gene encodes for the 24‐hydroxylase enzyme, which is responsible for the catabolism of 25‐hydroxyvitamin D (25(OH)D) and 1,25‐dihydroxyvitamin D (1,25(OH)2D). Mutations in *CYP24A1* can result in elevated 1,25(OH)2D causing parathyroid hormone (PTH)‐independent hypercalcemia, hypercalciuria, nephrolithiasis, and nephrocalcinosis. We present the cases of two siblings exhibiting hypercalcemia secondary to a *CYP24A1* loss‐of‐function mutation. Case 1 presented initially with PTH‐dependent hypercalcemia, with localization of a left upper parathyroid adenoma on parathyroid technetium sestamibi (^99m^Tc‐MIBI) uptake study. Despite parathyroidectomy (180 mg adenoma), hypercalcemia, hypercalciuria, and low normal PTH levels persisted. A repeat parathyroid ^99m^Tc‐MIBI uptake study localized a second adenoma and a right inferior parathyroidectomy was performed (170 mg adenoma). PTH subsequently became undetectable, however hypercalcemia and hypercalciuria persisted. A new presentation of PTH‐independent hypercalcemia found to be secondary to a *CYP24A1* loss‐of‐function mutation in his sibling, Case 2, signaled the underlying cause. Cascade testing confirmed both siblings were homozygous for the pathogenic variant c.1186C>T, p.Arg396Trp (R396W) of *CYP24A1* (NM_000782.5). In clinical practice *CYP24A1* loss‐of‐function mutations should be considered in patients presenting with PTH‐independent hypercalcemia, hypercalciuria, and 1,25(OH)2D levels in the upper normal or elevated range. Although in our case assays of 24,25(OH)2D were not available, calculation of the 25(OH)D:24,25(OH)2D ratio can assist in the diagnostic process. Possible treatments to manage the risk of hypercalcemia in patients with a *CYP24A1* loss‐of‐function mutation include avoidance of vitamin D oversupplementation and excessive sun exposure. Hydration and bisphosphonate therapy can be useful in managing the hypercalcemia. Although not utilized in our cases, treatment with ketoconazole, fluconazole, and rifampicin have been described as potential therapeutic options. © 2023 The Authors. *JBMR Plus* published by Wiley Periodicals LLC on behalf of American Society for Bone and Mineral Research.

## Graphical abstract

Hypervitaminosis D secondary to a *CYP24A1* loss of‐function mutation: an unusual cause of hypercalcaemia in two siblings.

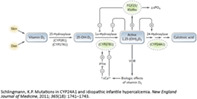

## Introduction

Hypervitaminosis D as a cause of hypercalcemia is often associated with low‐to‐undetectable parathyroid hormone (PTH) levels and may be due to vitamin D intoxication, granulomatous diseases, or abnormalities of vitamin D metabolism. The production of 1,25‐dihydroxyvitamin D (1,25(OH)2D), the active form of vitamin D, is mediated by 1α‐hydroxylase (*CYP27B1*). 25‐Hydroxyvitamin D (25(OH)D) undergoes hydroxylation via 1α‐hydroxylase to yield 1,25(OH)2D. Both 25(OH)D and 1,25(OH)2D are inactivated by 24‐hydroxylase (*CYP24A1*).^(^ ^1^ ^)^ Loss‐of‐function mutations in *CYP24A1* can result in elevated concentrations of 1,25(OH)2D causing hypercalcemia, hypercalciuria, low/undetectable PTH, nephrolithiasis, and nephrocalcinosis in children and adults.

*CYP24A1* loss‐of‐function mutations were first reported in 2011 as an underlying cause of idiopathic infantile hypercalcemia.^(^ ^2^ ^)^ Cases affecting both children and adults are well documented, with significant phenotypic variability.^(^ ^3^ ^)^

Here, we present the cases of two siblings exhibiting hypercalcemia secondary to a *CYP24A1* loss‐of‐function mutation. The diagnosis was complicated by the simultaneous occurrence of primary hyperparathyroidism in Case 1. Concurrent primary hyperparathyroidism and a *CYP24A1* loss‐of‐function mutation is very rare and of unclear mechanistic association.

## Patients and Methods

Genomic DNA was extracted from ethylenediamine tetraacetic acid (EDTA)‐anticoagulated peripheral whole blood using Qiagen Gentra® Puregene® Blood Core Kit B (QIAGEN, Valencia, CA, USA) for all patients.

The custom primer sets and experimental conditions for polymerase chain reaction (PCR) and bidirectional Sanger sequencing of the 11 protein‐coding exons (and their flanking intronic regions) of human *CYP24A1* were based on primer sequences published by Tebben and colleagues.^(^ ^4^ ^)^

All sequences were compared to the international reference DNA sequence of the human *CYP24A1* ([www.ncbi.nlm.nih.gov] NM_000782.5). Samples identified as containing deleterious variants by in silico tools had their sequence compared to previously reported variants (ClinVar [www.ncbi.nlm.nih.gov/clinvar] and Genome Aggregation Database (gnomAD) [https://gnomad.broadinstitute.org]), and variant nomenclature validated as per Human Genome Variation Society (HGVS) recommendations.^(^ ^5^ ^)^

Clinical samples, where pathogenic variants had been identified, had the presence of those variants confirmed with testing performed on second samples (data not shown).

## Case Report

### Case 1

A 53‐year‐old man was investigated for symptomatic hypercalcemia. His medical history included hypertrophic cardiomyopathy with previous septal myomectomy, paroxysmal atrial fibrillation, ischemic heart disease, and obstructive sleep apnea. He denied history of nephrolithiasis, hypercalcemia, and failure to thrive as a child and minimal trauma fractures. He was not taking regular vitamin D or calcium supplementation and his dietary calcium intake was adequate. Corrected calcium (calculated as total calcium (mmol/L) + [(40 − albumin (g/L)) × 0.02] was 3.08 mmol/L (laboratory reference range, 2.15–2.65), PTH 2.5 pmol/L (1.5–7.6; Siemens Atellica immunoassay; Siemens Medical Solutions USA, Inc., Malvern, PA, USA), phosphate 1.0 mmol/L (0.8–1.4), creatinine 102 μmol/L (45–90), 25(OH)D 52 nmol/L (>50; DiaSorin Liaison XL immunoassay; DiaSorin, Stillwater, MN, USA), and 1,25(OH)2D 330 pmol/L (78–190; DiaSorin Liaison XL immunoassay). Urine calcium excretion was 15.9 mmol/24 hours (2.0–7.5). Serum angiotensin‐converting enzyme (ACE) was normal and serum and urine monoclonal proteins were absent. Chest X‐ray and whole‐body bone scan were unremarkable. Renal ultrasonography revealed no evidence of nephrolithiasis or nephrocalcinosis. A parathyroid ^99m^Tc‐MIBI uptake study led to a focused left upper parathyroidectomy. An 180‐mg adenoma was resected with histologic features consistent with parathyroid adenoma.

Hypercalcemia, hypercalciuria, with low normal PTH levels persisted postoperatively. The patient was unwilling to undergo multiple endocrine neoplasia (MEN) genetic testing. Bone mineral density (BMD) measurement displayed the following: total lumbar spine (L_1_–L_4_) *T* score −1.8, femoral neck *T* score −1.0, and distal radius *T* score −2.6 (Hologic Horizon; Hologic, Inc., Marlborough, MA, USA). A computed tomography (CT) scan of his chest, abdomen, and pelvis was performed. A 4‐mm calcified granuloma was noted in the middle lobe of the right lung. This finding led to the completion of an fluorodeoxyglucose–positron emission tomography (^18^FDG‐PET)/CT study that was unremarkable. Due to the incidental finding of a small granuloma, a trial of prednisolone was undertaken. No change in ionized calcium or urinary calcium excretion was noted. A positive parathyroid ^99m^Tc‐MIBI uptake study led to a right inferior parathyroidectomy (170‐mg adenoma). After the second parathyroidectomy, hypercalcemia (2.86 mmol/L), hypercalciuria (8.3 mmol/24 hours) persisted but PTH became undetectable (<0.3 pmol/L) and 1,25(OH)2D was 130 pmol/L.

A new presentation with PTH‐independent hypercalcemia in a sibling (Case 2) signaled the underlying cause.

### Case 2

A 62‐year‐old woman presented with PTH‐independent hypercalcemia during hospitalization for a subarachnoid hemorrhage. Her medical history included diet‐controlled type 2 diabetes mellitus, fibromyalgia, anxiety, and depression. She had a distant history of nephrolithiasis and a family history of hypercalcemia in her brother (Case 1). She denied history of minimal trauma fractures and hypercalcemia and failure to thrive as a child. She was not taking regular vitamin D or calcium supplements and her dietary calcium intake was adequate. Corrected calcium was 2.96 mmol/L (2.15–2.65), PTH 0.5 pmol/L (1.5–7.6; Siemens Atellica immunoassay), phosphate 1.02 mmol/L (0.75–1.5), creatinine 61 μmol/L (40–90), 25(OH)D 76 nmol/L (>50; DiaSorin Liaison XL immunoassay), and 1,25(OH)2D 179 pmol/L (50–190; DiaSorin Liaison XL immunoassay). Urinary calcium excretion was 10.3 mmol/24 hours (2.0–7.5). Serum ACE was 46.1 U/L (8.0–75), PTH‐related protein <2.0 pmol/L (<2.0; Beckman Coulter immunoradiometric assay; Beckman Coulter, Brea, CA, USA) and serum and urine monoclonal proteins were absent. Chest X‐ray and whole‐body bone scan were unremarkable. Renal ultrasonography demonstrated no evidence of nephrolithiasis or nephrocalcinosis. BMD measurement showed the following: lumbar spine (L_1_–L_4_) *T* score −3.6, femoral neck *T* score −1.8, and distal radius *T* score −4.1 (Hologic Horizon).

Due to the absence of available assays for 24,25(OH)2 levels, genetic testing was undertaken. Sanger sequencing found her to be homozygous for the pathogenic variant c.1186C>T, p.Arg396Trp (R396W) in *CYP24A1* (NM_000782.5) known to cause loss‐of‐function.

Cascade testing identified the same mutation in her brother (Case 1).

## Discussion

Cytochrome‐P450‐24 subfamily A member 1 (*CYP24A1*) gene encodes for the 24‐hydroxylase enzyme responsible for the catabolism of 25(OH)D and 1,25(OH)2D.^(^ ^1^ ^)^ Loss‐of‐function mutations in *CYP24A1* can result in elevated concentrations of 1,25(OH)2D causing hypercalcemia, hypercalciuria, low/undetectable PTH, nephrolithiasis, and nephrocalcinosis in children and adults. *CYP24A1* loss‐of‐function mutations were first reported in 2011 as an underlying cause of idiopathic infantile hypercalcemia in 10 pediatric patients from nine families of German, Turkish, and Russian origin presenting with failure to thrive, vomiting, dehydration, and nephrolithiasis/nephrocalcinosis.^(^ ^2^ ^)^ The clinically relevant hypercalcemia was noted following intake of vitamin D as prophylaxis for rickets. Although the majority of documented cases in the literature are consistent with an autosomal recessive inheritance, autosomal dominant inheritance with incomplete penetrance have sporadically been postulated.^(^ ^4^ ^)^

Vitamin D undergoes hydroxylation by 25‐hydroxylase (*CYP2R1*) produced in the liver, yielding 25(OH)D. 1α‐Hydroxylase (*CYP27B1*) in the kidney catalyzes the second hydroxylation to yield active 1,25(OH)2D, which binds to the vitamin D receptor to exert biologic effect. 25(OH)D is catabolized to inactive 24,25(OH)2D and 1,25(OH)2D is converted, via a five‐step oxidation pathway, to metabolically inactive calcitroic acid by 24‐hydroxylase (*CYP24A1*). The biologic activity of *CYP27B1* and *CYP24A1* are regulated by serum calcium, 1,25(OH)2D levels, PTH, and fibroblast growth factor 23 (FGF23).^(^ ^1^, ^2^ ^)^

The importance of *CYP24A1* in the maintenance of 1,25(OH)2D and calcium homeostasis has been displayed in *CYP24A1* knockout mice (−/−) who experienced marked hypercalcemia. A delay in the clearance of 1,25(OH)2D was seen following administration of exogenous 25(OH)D and 1,25(OH)2D to *CYP24A1* −/− mice highlighting an intrinsic fault in the catabolism of 1,25(OH)2D.^(^ ^6^ ^)^

In clinical practice *CYP24A1* loss‐of‐function mutations should be considered in patients presenting with PTH‐independent hypercalcemia, hypercalciuria, and 1,25(OH)2D levels in the upper normal or elevated range. Although in our case assays of 24,25(OH)2D were not available, calculation of the 25(OH)D:24,25(OH)2D ratio can assist in the diagnostic process. In unaffected individuals, the levels of 25(OH)D and 24,25(OH)2D are proportional, and the ratio is typically less than 25.^(^ ^7^, ^8^ ^)^ However, in individuals harboring a *CYP24A1* loss‐of‐function mutation, 25(OH)D is elevated in comparison to 24,25(OH)2D, leading to an increased ratio, typically greater than 80.^(^ ^7^, ^8^ ^)^ Individuals with confirmed biallelic mutations have an elevated ratio when compared to patients with monoallelic variants and wild‐type controls.^(^ ^9^ ^)^

A mechanistic connection between primary hyperparathyroidism and *CYP24A1* loss‐of‐function mutations is not well understood nor well described in the literature. Similar to our case, isolated case reports have identified both hyperplastic parathyroid glands and discrete parathyroid adenomas in patients also harboring a *CYP24A1* loss‐of‐function mutation. It is not clear whether there is an underlying association between these two conditions, or merely a random coincidence.^(^ ^10^, ^11^ ^)^

Last, possible treatments to manage the risk of hypercalcemia in patients with a *CYP24A1* loss‐of‐function mutation include avoidance of vitamin D oversupplementation and excessive sun exposure. Hydration and bisphosphonate therapy can be useful in managing the hypercalcemia. In addition, bisphosphonate therapy can improve the low bone mineral density associated with a *CYP24A1* loss‐of‐function mutation. Excessive osteoclastic bone resorption mediated by elevated 1,25(OH)2D levels has been postulated as a cause of low bone mass.^(^ ^12^, ^13^ ^)^ Both of our cases required bisphosphonate therapy to manage their hypercalcemia and low BMD. Although not utilized in our cases, treatment with ketoconazole, fluconazole, or rifampicin has been described as potential therapeutic options. Ketoconazole or fluconazole inhibit *CYP27B1*, which is involved in the hydroxylation of 25(OH)D to 1,25(OH)D. Undesirably, ketoconazole or fluconazole can also inhibit *CYP3A4*, thereby theoretically reducing the degradation of several vitamin D metabolites, including 25(OH)D and 1,25(OH)2D. Rifampicin, on the other hand, induces *CYP3A4*, leading to acceleration in the degradation of 25(OH)D and 1,25(OH)2D. Rifampicin has been utilized effectively to manage hypercalcemia, nephrolithiasis, and nephrocalcinosis in 1‐year and 2‐year experiences.^(^ ^14^, ^15^ ^)^ Hawkes and colleagues^(^ ^14^ ^)^ displayed improvement in hypercalcemia, hypercalciuria, and nephrocalcinosis in two patients during a 13‐month follow‐up. These findings were corroborated by Brancatella and colleagues^(^ ^15^ ^)^ over a 24‐month treatment course, with reported side effects of rifampicin including daytime sleepiness and asthenia, without impairment of liver function, total blood count, or adrenal function. Overall, the long‐term efficacy and safety of ketoconazole, fluconazole, or rifampicin for this purpose, however, remains to be established.^(^ ^4^, ^14^ ^)^

This case report highlights a clinically relevant condition that ought to be considered in patients presenting with PTH‐independent hypercalcemia, hypercalciuria, and 1,25(OH)2D levels in the upper normal or elevated range. In addition, the association between *CYP24A1* loss‐of‐function mutations and nephrolithiasis and/or nephrocalcinosis is apparent and should be considered in patients presenting with recurrent renal calcium deposition.^(^ ^4^, ^10^, ^16^ ^)^

## Author Contributions

**Lucy Collins:** Writing – original draft; writing – review and editing. **Emma Boehm:** Writing – review and editing. **Catherine Luxford:** Writing – review and editing. **Roderick J. Clifton‐Bligh:** Writing – review and editing. **Vivian Grill:** Supervision; writing – original draft; writing – review and editing.

## Disclosures

The authors declare there are no competing financial interests or conflicts of interest.

### Peer Review

The peer review history for this article is available at https://www.webofscience.com/api/gateway/wos/peer‐review/10.1002/jbm4.10788.

## Data Availability

Data sharing is not applicable to this article as no datasets were generated or analysed during the current study.
